# Multiple trait association analysis revealed common genetic loci between lung cancer and heart failure

**DOI:** 10.1186/s40959-025-00438-y

**Published:** 2026-01-12

**Authors:** Linquan Mu, Yi zhou, Songpu Li, Feng Liu

**Affiliations:** 1 The Second People’s Hospital of Yibin, Yibin, 644000 China; 2 Nanjing Medical University, Nanjing, 211166 China; 3 Intelligent Information Technology Research Center, Jingchu University of Techology, Jingmen, 448000 China; 4 Jingmen Cryptometry Application Techology Research Center, Jingmen, 448000 China; 5 Key Laboratory of Medical Electrophysiology, Ministry of Education and Medical Electrophysiological Key Laboratory of Sichuan Province, Collaborative Innovation Center for Prevention of Cardiovascular Diseases, Institute of Cardiovascular Research, Southwest Medical University, Luzhou, China; 6 Department of Cardiovascular Surgery, The Affiliated Hospital of Southwest Medical University, Luzhou, 646000 China; 7 Cardiovascular Remodeling and Dysfunction Key Laboratory of Luzhou, Luzhou, 646000 China

**Keywords:** Lung cancer, Heart failure, Genetic correlation, Neural signaling, Single-cell analysis

## Abstract

**Background:**

Lung cancer (LC) and heart failure (HF) frequently co-occur with substantial clinical consequences, yet their shared genetic architecture remains poorly characterized. Emerging evidence suggests common pathophysiological pathways may underlie this comorbidity, particularly involving neural signaling and inflammatory processes.

**Methods:**

We conducted cross-trait meta-analyses of genome-wide association studies (GWAS) encompassing 23 cancer types and 14 cardiovascular diseases using MTAG and CPASSOC. Tissue-specific expression patterns were evaluated using GTEx data, while single-cell RNA sequencing analyzed differential gene expression in HF patients and LC cases compared to healthy controls. Pharmacological screening was performed using DrugBank and PharmGKB databases to identify potential therapeutic candidates.

**Results:**

Our analyses identified 48 pleiotropic single nucleotide polymorphisms clustered within chromosome 15q25.1 that were significantly associated with both LC and HF (*p* < 1 × 10^− 76^). These variants implicated key genes including *CHRNA3*, *CHRNA5*, *HYKK*, and *PSMA4*, which demonstrated enrichment in neuroactive ligand-receptor interaction pathways and specific expression in cardiac tissues and immune cells. Twenty candidate drugs targeting cholinergic pathways were identified.

**Conclusions:**

These findings uncover a shared genetic locus and neural pathways underlying LC-HF comorbidity, offering mechanistic insights and therapeutic opportunities. The results highlight the need for integrated cardiology-oncology approaches in managing these complex conditions.

**Graphical Abstract:**

Graphical overview of the integrative pipeline employed to uncover the shared genetic architecture between lung cancer and heart failure. The workflow sequentially applies genome-wide LD-score regression to estimate genetic correlation, cross-trait analyses to pinpoint pleiotropic loci, gene- and pathway-level enrichment to highlight convergent biology, tissue-specific eQTL colocalization to map regulatory effects, cross-phenotype network interrogation to reveal broader disease connections, and drug–target matching to identify repurposing opportunities, culminating in a PheWAS summary that contextualizes the clinical relevance of the identified variants

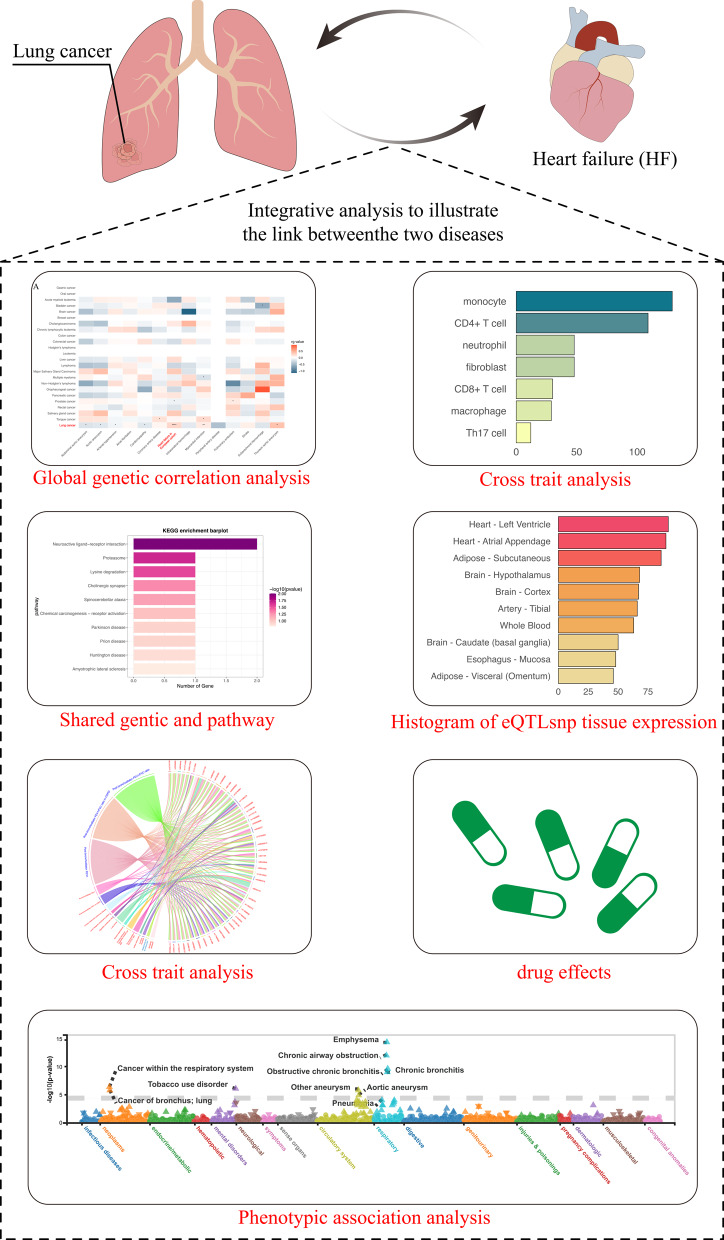

**Supplementary Information:**

The online version contains supplementary material available at 10.1186/s40959-025-00438-y.

## Introduction

Cancer and cardiovascular diseases (CVDs) are among the most prevalent and fatal diseases worldwide [1], with overlapping risk factors—including hypertension, obesity, smoking, and metabolic disorders—underscoring their interrelated pathophysiology [2–4]. Emerging evidence demonstrates bidirectional risk escalation: cancer survivors face elevated CVD incidence, while CVD patients exhibit higher cancer susceptibility [5–9].

Among these, lung cancer (LC) and heart failure (HF) are particularly lethal. LC remains the most prevalent cancer-related death [10], whereas HF, the end-stage manifestation of CVDs, imposes severe morbidity and mortality [11]. Clinically, LC-HF comorbidity is frequent and exacerbates patient outcomes [12–15], yet the shared genetic architecture remains poorly characterized. Both diseases show significant heritability (HF: ~26%; LC: ~13.7%) [16, 17], and recent GWAS have identified risk loci for each [18–21]. Notably, genetic predisposition to LC correlates with HF development [12], suggesting common mechanistic pathways. Despite extensive clinical evidence highlighting the common occurrence of LC and HF, the shared genetic etiology between these conditions remains not fully understood. Genome-wide association studies (GWAS), particularly those involving large sample studies conducted in recent years, have identified single nucleotide variants (SNVs) associated with many complex diseases [22]. These studies have significantly expanded the catalog of HF- and LC-related loci compared to earlier research, greatly enhancing our understanding of these conditions [21, 23, 24].

Here, we systematically analyzed large-scale GWAS data to delineate shared genetic variants between LC and HF. We identified 48 pleiotropic SNPs, with colocalization implicating *CHRNA3*, *CHRNA5*, *HYKK*, and *PSMA4* as key loci. Single-cell transcriptomics validated these associations, while enrichment analyses highlighted roles in neurotransmitter signaling and metabolic regulation. Tissue-specific signals localized to cardiac (left ventricle, appendage) and subcutaneous adipose tissues, with cellular enrichment in monocytes and CD4 + T cells. Pharmacogenetic screening further revealed candidate therapeutics targeting LC, neuroregulation, and hypertension. Our findings elucidate genetic convergence between LC and HF, offering translational opportunities for dual-pathway interventions.

## Results

### GWAS datasets and quality control

**Fig. 1:**
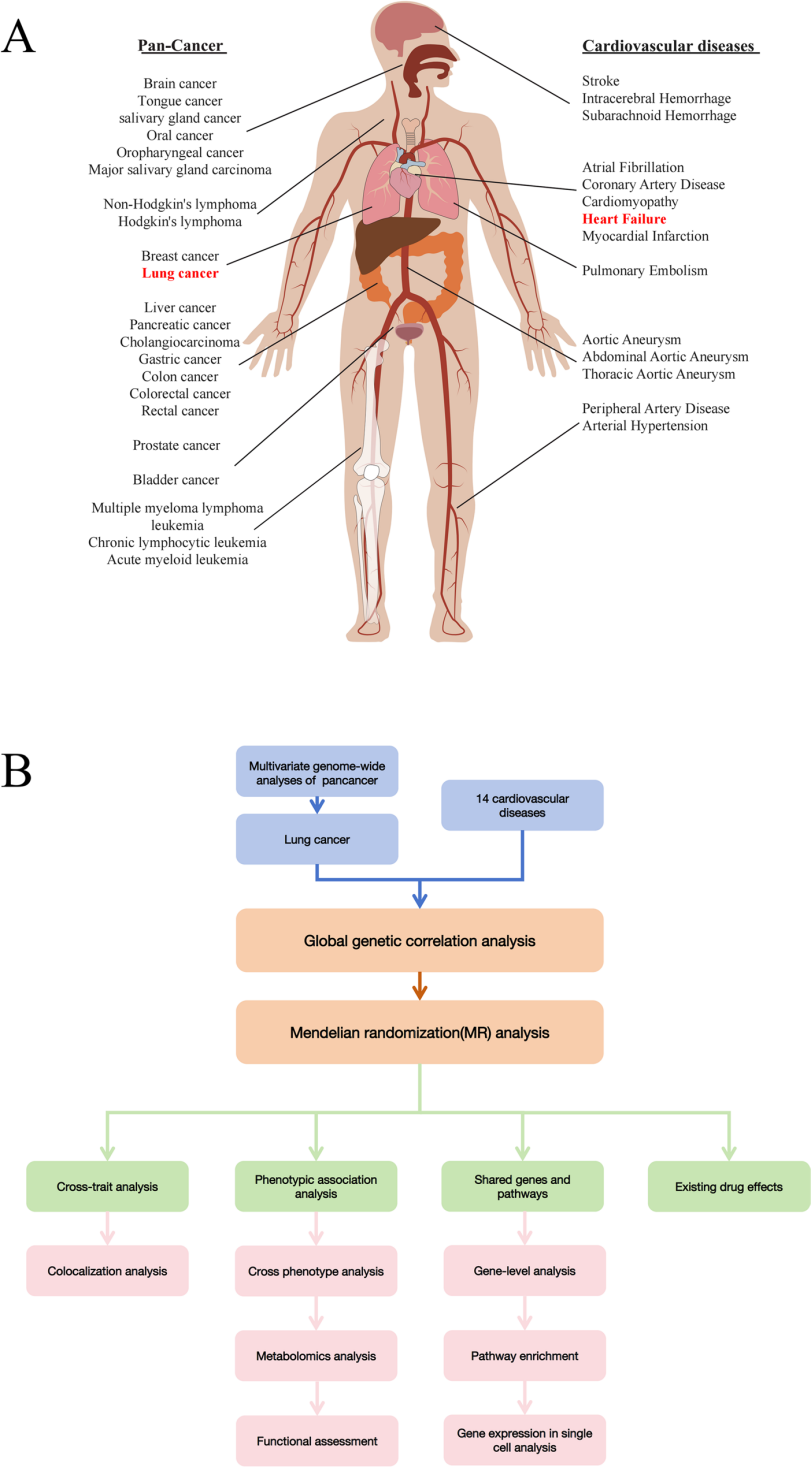
Interrogation of genetic components between pancancer and cardiovascular diseases. **A** Traits include pan-cancer and cardiovascular diseases. **B** Overview of our study

Our analysis incorporated comprehensive GWAS data from 23 cancer types (including histological and anatomical subtypes) and 14 cardiovascular diseases (Fig. 1A). The cancer datasets, derived from European populations, encompassed a broad spectrum of malignancies: hematologic (e.g., leukemia, lymphoma), solid tumors (e.g., lung, breast, colorectal cancers), and rare neoplasms (e.g., salivary gland, cholangiocarcinoma). All cancer datasets maintained rigorous quality thresholds, with sample sizes > 40,000 individuals and > 3 million single nucleotide variants (SNVs) per study.

The cardiovascular datasets similarly represented European populations and covered four major CVD categories: cerebrovascular diseases, thrombotic disorders, aortic aneurysms, and other cardiovascular conditions. These datasets predominantly originated from leading consortiums including CARDIoGRAM, METASTROKE, UK Biobank, and the AFGen Consortium, with most studies exceeding 300,000 participants and 3 million SNVs.

To systematically interrogate the shared genetic architecture between cancers and CVDs, we developed an integrative analytical framework (Fig. 1B) that simultaneously evaluates: [1] common SNVs exhibiting pleiotropic effects [2], shared susceptibility genes [3], convergent biological pathways [4], tissue-specific expression patterns, and [5] cell-type enrichment profiles across disease spectra.

### Genetic correlation analysis

We performed genome-wide genetic correlation analysis between 23 cancer types and 14 cardiovascular diseases (CVDs) using linkage disequilibrium score regression (LDSC) [25]. The most prominent findings revealed significant positive genetic correlations between lung cancer subtypes and heart failure (HF). Notably, the overall genetic correlation between lung cancer and HF was particularly strong in European populations (rg = 0.2352, *p* = 1.4434e^− 05^), with small cell lung carcinoma (rg = 0.228, *p* = 0.0142) and squamous cell lung carcinoma (rg = 0.1638, *p* = 0.0223) showing consistent positive associations. This relationship was further validated in multi-ethnic cohorts, which included 5,791 African ancestry cases, 20,883 African ancestry controls, 1,170 admixed ancestry cases, 13,217 admixed ancestry controls, 12,665 East Asian ancestry cases, 245,263 East Asian ancestry controls, 95,524 European ancestry cases, and 1,270,968 European ancestry controls. These cohorts spanned various countries, including 26,674 African ancestry participants (U.S., NR), 14,387 other admixed ancestry participants (NR), 257,928 East Asian ancestry participants (NR, Japan, China), and 1,366,492 European ancestry participants (U.S., Finland, U.K.). The analysis in these multi-ethnic cohorts(rg = 0.2276, *p* = 1.6562e^− 05^)further reinforced the shared genetic basis between lung cancer and heart failure.

**Fig. 2:**
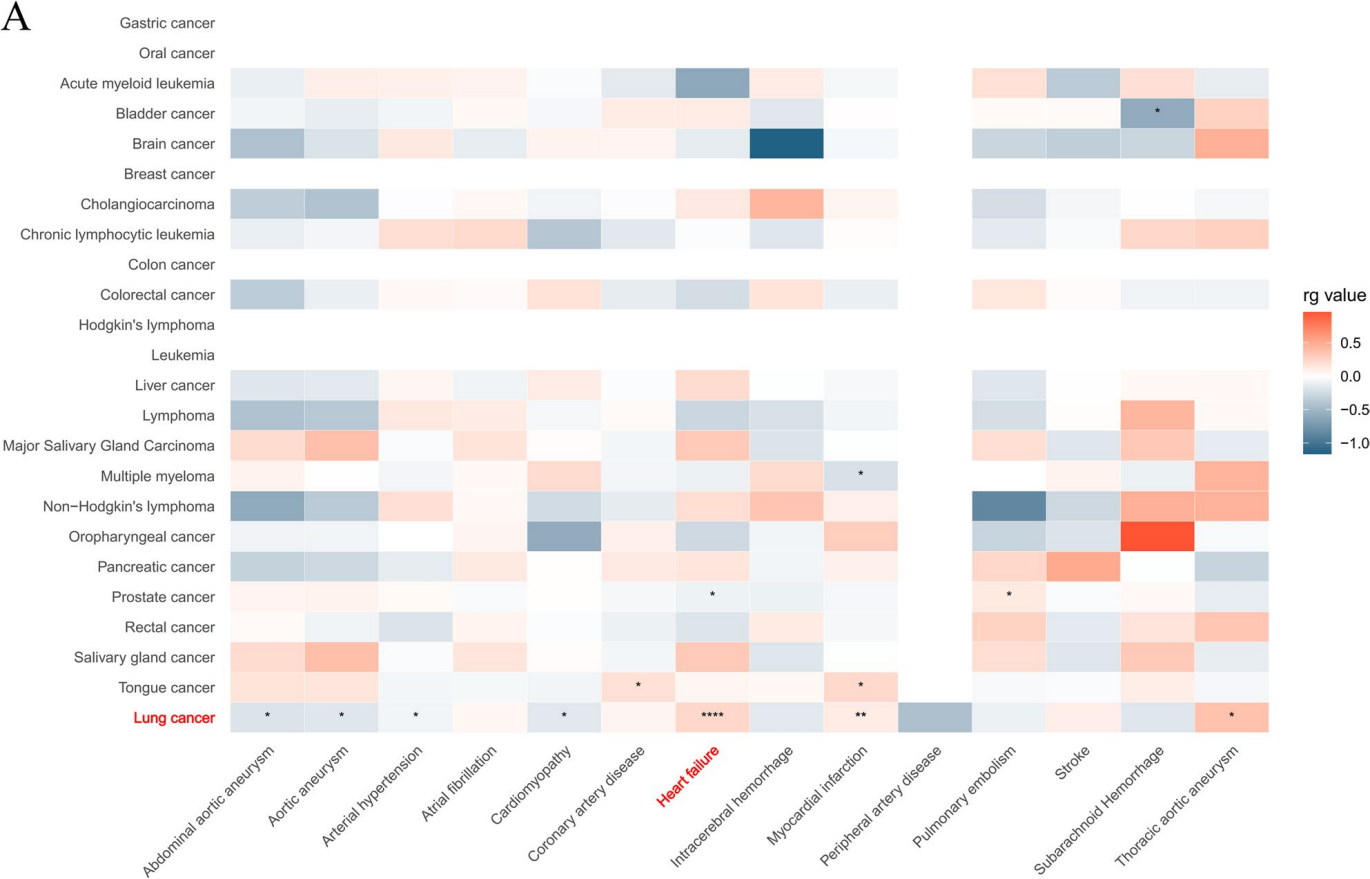
Genetic correlation between pancancer and cardiovascular diseases. Heatmap of genetic correlation (rg) calculated by LDSC for 23 cancers and 14 cardiovascular diseases

Additionally, small cell lung carcinoma exhibited a positive correlation with myocardial infarction (rg = 0.1703, *p* = 0.0018), while prostate cancer demonstrated differential associations—a weak negative correlation with HF (rg = −0.0909, *p* = 0.0128) but a positive correlation with pulmonary embolism (rg = 0.1267, *p* = 0.0119). Collectively, these results, detailed in Fig. 2 and Table S1, identify eight significant trait pairs linking lung cancer and HF, highlighting their potential shared genetic etiology. The robust and reproducible associations across populations suggest that common genetic mechanisms may contribute to the comorbidity of lung cancer and cardiovascular diseases.

### Causal inference analysis

To elucidate the potential causal relationship between lung carcinoma (LC) and heart failure (HF), we conducted bidirectional Mendelian randomization (MR) analyses using genetic variants as instrumental variables. This approach addresses limitations inherent in observational studies that have previously examined associations between LC, HF, and related metabolic factors (e.g., BMI, HDL). Our comprehensive analysis, incorporating multiple MR models (Table S2), consistently demonstrated absence of causal effects in either direction: HF as exposure showed no significant effect on LC risk (IVW *β* = −0.049, *p* = 0.688), nor did LC as exposure influence HF development (IVW *β* = −0.042, *p* = 0.688). These robust null findings suggest that the well-documented epidemiological associations between LC and HF likely arise from shared biological pathways or confounding factors rather than direct causal mechanisms. The results provide important clarification for understanding the complex interplay between these conditions, indicating that their frequent co-occurrence may be driven by common underlying risk factors rather than causative relationships.

### Identification of shared genetic variants between LC and HF

Through cross-trait meta-analysis integrating MTAG (Multi-Trait Analysis of GWAS) [26] and CPASSOC (Cross-Phenotype Association Analysis) methodologies [27], we identified 48 SNVs significantly shared between LC and HF. These pleiotropic variants met stringent significance thresholds (PMTAG & PCPASSOC) < 5 × 10^− 8^ and were further validated by cross-trait colocalization analysis [28]. Remarkably, all the shared 48 SNVs clustered within a 196-kb region on chromosome 15q (positions 78,730,313 − 78,926,445), exhibiting high statistical significance(*P* < 1 × 10^− 76^), as shown in Table S3. This genomic region has been previously implicated in both congenital heart disorders and lung cancer, particularly through the CHRNA5–CHRNA3 locus, thereby providing independent support for our findings [29–31].

**Fig. 3:**
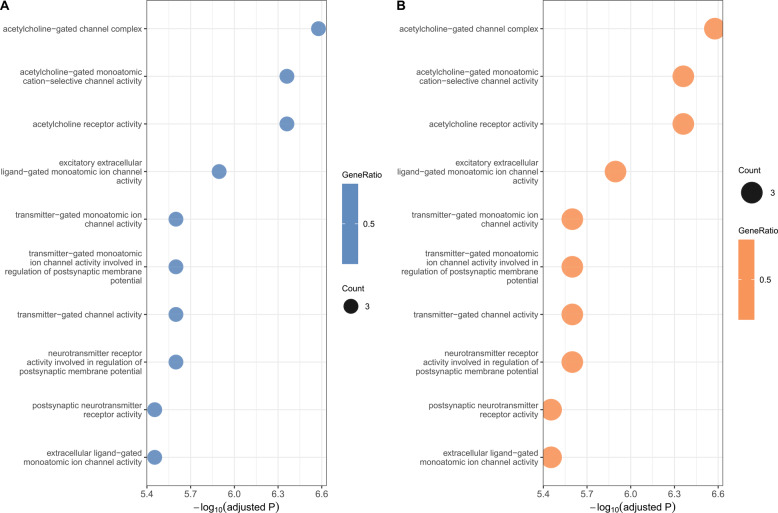
Functional annotation results of shared genes between LC and HF. **A** GO pathway enrichment of the shared genes between LC and HF, categorized by SNV location. **B** B KEGG pathway enrichment of the shared genes between LC and HF, categorized by SNV location

Gene mapping revealed these variants primarily localize to key functional genes: *IREB2* (16 SNVs), *CHRNA5* [12], *HYKK* [9], *CHRNA3* [6], with additional variants in *CHRNB4*, *PSMA4*, and intergenic regions. Functional annotation of 45 SNVs through Gene Ontology analysis (GO) demonstrated significant enrichment for neurotransmission and synaptic activity processes (Fig. 3A).Parallel Kyoto Encyclopedia of Genes and Genomes (KEGG) pathway analysis of 29 SNVs highlighted involvement in neuroactive ligand-receptor interactions and lysine degradation pathways (Fig. 3B).These results collectively identify chromosome 15q as a critical genomic locus contributing to the shared genetic architecture of LC and HF, with neurotransmission-related pathways emerging as potential mechanistic links between these conditions.

### Phenotypic association analysis of shared genetic variants

**Fig. 4:**
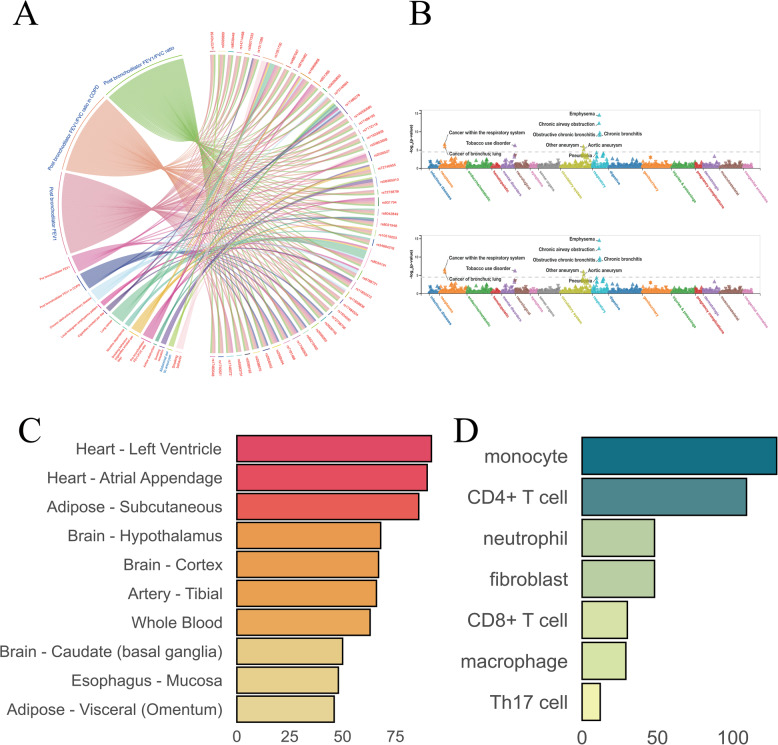
Functional annotation results of shared genes between LC and HF. **A** Shared SNVs with genome-wide significant loci in the GWAS catalog. **B** Two representative SNVs with strongly associated signal loci in PheWeb. **C** Tissue regions enriched in expression for eQTLs of shared SNVs. **D** Cellular regions enriched in expression for eQTLs of shared SNVs

Through systematic interrogation of GWAS Catalog data [32], we characterized phenotypic associations of the 48 identified SNVs at genome-wide significance(*P* < 5 × 10^− 8^). Strikingly, all variants demonstrated association with post-bronchodilator FEV1 measures, while subsets showed significant links to lung cancer (8/48 SNVs) and chronic obstructive pulmonary disease (COPD) (Fig. 4A). PheWeb-based enrichment analysis further revealed these variants to be overrepresented in respiratory, circulatory, and neoplastic disease categories (Fig. 4B), though metabolite analysis in METSIM and mGWAS-Explorer yielded no significant associations.To elucidate functional mechanisms, we performed comprehensive expression quantitative trait locus (eQTL) mapping using the Genotype-Tissue Expression (GTEx) database and xQTLbiolinks platform. The variants exhibited pronounced tissue-specific enrichment in cardiac (left ventricle and atrial appendage) and subcutaneous adipose tissues (Fig. 4C), with cellular-level analyses identifying monocytes and CD4^+^ T cells as key immune cell populations harboring these associations (Fig. 4D). These findings collectively position the identified SNVs at the intersection of pulmonary function, cardiovascular biology, and immune regulation, providing mechanistic insights into their pleiotropic effects across organ systems.

### Integrative functional genomics reveals shared pathways between LC and HF

Conventional proximity-based gene annotation methods often overlook the complex pleiotropic effects of GWAS variants. To overcome this limitation, we implemented a tripartite analytical approach integrating TWAS-Fusion [33], SMR [34], and MAGMA [35] methods. This robust convergence analysis identified four core genes: *CHRNA3*, *CHRNA5*, *HYKK*, and *PSMA4*, which are consistently associated with both phenotypes across all methodologies.

**Fig. 5:**
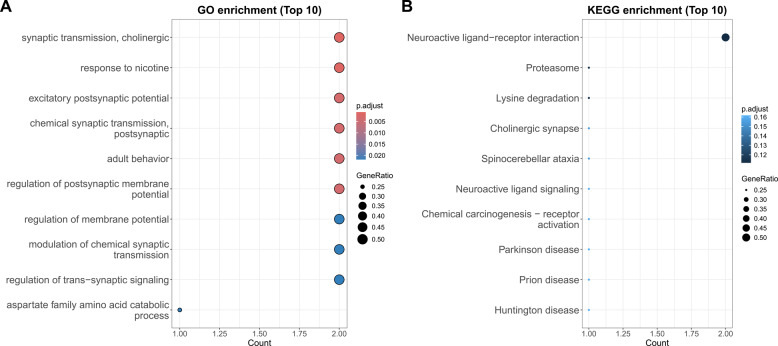
Functional annotation results of comorbid risk genes. **A** GO pathway enrichment of the comorbid risk genes. **B** KEGG pathway enrichment of comorbid risk genes

GO enrichment analysis demonstrated significant overrepresentation of terms related to neural signaling and synaptic function. The most highly enriched cellular component terms included the acetylcholine-gated channel complex (FDR = 9.1 × 10^− 5^), postsynaptic membrane (FDR = 8.8 × 10^− 3^), and monoatomic ion channel complex (FDR = 8.8 × 10^− 3^), suggesting a strong association with cholinergic neurotransmission and neuronal excitability(Fig. 5A, Table S4). Complementary KEGG analysis demonstrated equally compelling results, with neuroactive ligand-receptor interactions emerging as the most significantly enriched pathway (*P* = 0.00976). These results collectively highlight the dual roles of these genes in neurotransmission and cellular metabolism (Fig. 5B).

These convergent findings from orthogonal analytical approaches establish *CHRNA3/CHRNA5-HYKK-PSMA4* as a functionally coherent gene network operating at the intersection of neuronal signaling and cardiopulmonary pathophysiology. The remarkable consistency across independent methodologies and functional databases strongly supports their biological relevance in mediating pleiotropic effects between LC and HF.

### Single-cell transcriptomic profiling reveals disease-specific expression patterns of comorbidity-associated genes

**Fig. 6:**
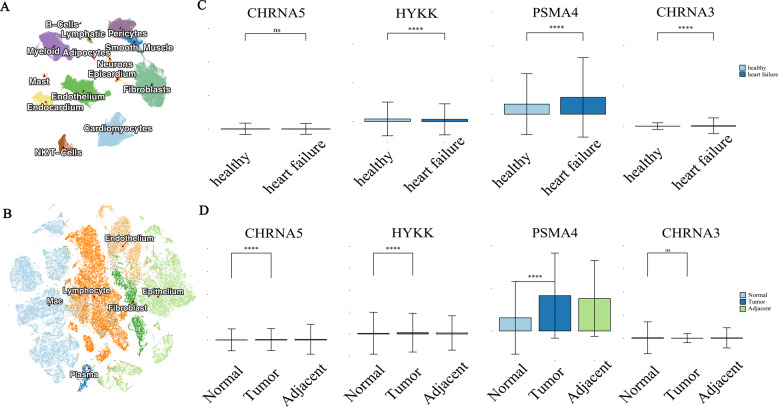
Single-cell RNA sequencing analysis reveals comorbid risk genes in HF and LC. **A** Umap plot illustrating the clustering of various cell types in HF. **B** tSNE plot illustrating the clustering of various cell types in LC. **C**-**D** Comorbid risk gene expressions in HF and LC, the first row represents the expression differences of four genes between HF and normal tissues. The second row represents the expression differences of the four genes between LC, adjacent non-cancerous tissue, and normal individuals

Building upon our genetic findings, we performed comprehensive single-cell resolution analyses to characterize the expression patterns of identified comorbidity risk genes (*CHRNA3*,* CHRNA5*,* HYKK*, and *PSMA4*) in both HF and LC. For HF, we analyzed a high-quality integrated dataset comprising 220,752 nuclei and 49,723 cells from 45 individuals (18 HF patients and 27 healthy controls), representing 15 major cardiac cell populations(Fig. 6A) [36]. Through rigorous quality control and harmonized processing using Seurat and Harmony pipelines, we established a robust cellular atlas encompassing fibroblasts, endothelial cells, immune subsets (myeloid cells, T cells, NK cells, B cells), and structural components (pericytes, smooth muscle cells).

While the cellular distribution patterns of comorbidity genes showed no significant variation across cell types, differential expression analysis revealed striking disease-associated dysregulation. In HF, *CHRNA3*, *HYKK*, and *PSMA4* exhibited marked expression differences between patients and controls (Wilcoxon test,*P* < 0.0001), with tissue-level aggregate scoring confirming these findings. Parallel analysis of LC specimens demonstrated similarly significant dysregulation of *CHRNA5*, *HYKK*, and *PSMA4* in malignant versus normal lung tissues (Wilcoxon *P* < 0.0001)(Fig. 6C-D). The consistent differential expression of these genes across both disease contexts, particularly in key cell populations, strengthens their potential role as molecular mediators of LC-HF comorbidity. These single-cell resolution findings complement our genetic analyses and provide mechanistic insights into how shared risk variants may influence disease pathophysiology through cell-type specific expression changes.The single-cell transcriptional profiling of lung cancer specimens, including malignant tissues, adjacent normal tissues, and healthy controls (Fig. 6B), revealed consistent dysregulation patterns mirroring our cardiac findings. Specifically, *CHRNA5*, *HYKK*, and *PSMA4* demonstrated highly significant differential expression between LC patients and healthy controls (Wilcoxon test, *P* < 0.0001). Tissue-level aggregation analysis using the AddModuleScore function further validated these expression differences (Wilcoxon test, *P* < 0.0001), confirming the robustness of our observations across analytical approaches.

The convergent dysregulation of these comorbidity-associated genes in both LC and HF contexts - with *CHRNA5/HYKK/PSMA4* in lung cancer and *CHRNA3/HYKK/PSMA4* in heart failure - suggests their potential role as molecular nodes linking these pathophysiologically distinct conditions. Notably, *HYKK* and *PSMA4* emerged as consistently dysregulated across both diseases, while the nicotinic receptor subunits showed disease-specific patterns (*CHRNA5* in LC versus *CHRNA3* in HF). This distinctive yet overlapping expression profile positions these genes as compelling candidates for therapeutic intervention in comorbid patients. The robust statistical significance (*P* < 0.0001) across all comparisons strengthens the biological plausibility of targeting this gene network to simultaneously modulate both disease processes, potentially offering a novel treatment strategy for this high-risk patient population.

### Drug for LC and HF

We found that drug targets related to LC and HF were significantly enriched for the candidate genes *CHRNA3*, *CHRNA5*, *HYKK*, and *PSMA4*. We referenced several large drug-gene databases in our analysis, including DrugCentral [37], DGIdb [38], PharmGKB [39], and DrugBank [40], which provided comprehensive data support. Based on their potential role in treating both LC and HF, we identified 20 candidate drugs (Table S5) that have shown promise in treating both LC and CVDs. These drugs are mainly divided into five functional categories, 12 in the cholinergic pathway, 3 in the complement system, 3 antihypertensive drugs, 1 related to cardiac electrophysiological functions, and 1 with unknown therapeutic effects. This classification not only highlights the diverse mechanisms of action but also offers clear directions for clinical application. The findings indicate that these drugs hold significant potential for treating comorbid LC and HF, offering a scientific basis for further clinical research and practice.

## Discussion

In recent years, increasing human life expectancy has led to a growing prevalence of comorbid cancer and CVDs, which has become a significant public health concern. While numerous studies have highlighted the comorbidity between various cancers and cardiovascular diseases [41, 42], our investigation revealed novel insights at the genetic level. Utilizing aggregate-level statistics from GWAS, we identified extensive and robust genetic correlations between LC and and a spectrum of CVDs. Notably, no significant genetic correlation was observed between prostate cancer and heart failure after applying the Bonferroni correction (*p* < 0.05/23), despite the uncorrected p-value = 0.0128. Similarly, a positive genetic correlation between prostate cancer and pulmonary embolism (*p* = 0.045) did not meet the corrected significance threshold. Additionally, negative associations were observed between multiple myeloma and myocardial infarction, and between bladder cancer and subarachnoid hemorrhage. These findings, detailed in Fig. 2 and Table S1, contradict the earlier statement suggesting no significant correlations for other cancer types analyzed. Among the strongest associations identified, LC and HF showed a significant positive correlation in both the UKB and HERME cohorts (rg = 0.2352, *p* = 1.4434e^− 05^; rg = 0.2276, *p* = 1.65620e^− 05^). Although inverse variance weighting (IVW) analysis did not show a significant causal effect between LC and HF, this association may be confounded by shared risk factors. Importantly, our findings are corroborated by a recent large-scale clinical trial from the UK Biobank cohort, which prospectively followed 455,804 participants free of lung cancer at baseline, and 478,756 participants free of cardiovascular disease (CVD). The study reported substantial comorbidity rates between lung cancer (LC) and heart failure (HF), but did not infer a causal link between the two conditions [12].

This study further analyzed the relationships between LC and HF, highlighting the multifaceted variations, genes, biological pathways, and traits that are shared among cell and tissue types. These findings provide deeper insights into the common genetic underpinnings of LC and HF. Notably, our analysis revealed that LC and HF share the highest number of SNVs (*N* = 48). Strikingly, all 48 shared SNVs cluster within a specific region on chromosome 15 (15q), precisely spanning genomic positions 78,730,313 to 78,926,445.This chromosomal locus is significant, as prior studies have consistently linked chromosome 15 abnormalities to both cardiovascular disease and lung cancer risk [29–31]. Furthermore, functional annotation indicated that these SNVs are predominantly active in the heart (left ventricle, atrial appendage) and subcutaneous adipose tissue. At the cellular level, significant enrichment was observed in monocytes and CD4^+^ T cells, suggesting potential cell-type-specific regulatory mechanisms.

Four genes (*CHRNA3*, *CHRNA5*, *HYKK*, and *PSMA4*) were mapped to the 15q25.1 locus via SNP annotation. These genes are known to affect nicotine dependence [43–45],with polymorphisms (e.g., rs1051730) conferring heightened susceptibility to LC, COPD, and peripheral vascular disease [46, 47]. Critically, their genetic variations correlate with both smoking behaviors and disease risk [48]. Furthermore, single-cell RNA sequencing of heart failure (HF) patients versus healthy controls revealed statistically significant dysregulation of *CHRNA3*, *HYKK*, and *PSMA4* (Wilcoxon test, *P* < 0.0001). Parallel analysis in LC tissues, adjacent non-tumor tissues, and normal lung tissues confirmed pronounced expression differences in these same genes between LC patients and healthy individuals (Wilcoxon test, *P* < 0.0001). This consistent dysregulation across both conditions implicates these genes as core mediators of LC-HF comorbidity.

Functional enrichment analysis highlighted the central involvement of these genes in neuronal signaling and synaptic transmission. This aligns with emerging evidence that neurotransmitters directly modulate tumor progression [49–52] and cardiovascular pathophysiology [53–55]. Notably, in silico drug screening revealed that multiple approved LC therapeutics also target neurotransmitter pathways relevant to CVDs and hypertension. This pharmacological overlap is mechanistically supported by recent findings demonstrating functional synapses between LC cells and neurons that facilitate metastasis and aggressiveness [56–58]. Importantly, several high-priority drug candidates identified here await clinical validation, underscoring their translational potential.

Clinically, this study makes three significant contributions: First, we establish *CHRNA3*,* HYKK*, and *PSMA4* as genetic drivers of LC-HF comorbidity by demonstrating their concordant dysregulation across germline, transcriptional, and functional levels. Second, we identify neurotransmitter and synaptic dysfunction as a central pathogenic pathway mechanistically linking both conditions. Finally, we pinpoint promising drug candidates with dual activity against LC and HF, which urgently warrant prioritized clinical investigation.

Our study presents several strengths. First, it complements epidemiological studies, providing a genomic perspective on the correlation between lung cancer and heart failure. Second, 48 SNVs were identified using multiple methods, which jointly contribute to the genetic basis of both diseases. Third, we explored how these SNVs influence gene expression involved in neural signaling and synaptic function. Finally, we identified a novel drug from existing medications, which could serve as a potential therapeutic target for treating both diseases.

Several constraints warrant acknowledgment. First, The exclusive reliance on GWAS data limited variant detection to common SNVs, precluding analysis of rare variants, indels, and structural variations. Second, Restricted genetic diversity in our predominantly European cohort may obscure population-specific risk variants. Third, While genetic variation was comprehensively examined, critical environmental modulators (particularly smoking-induced epigenetic modifications) [59] were not systematically evaluated. Fourth, Mechanistic interpretations were constrained by current reference databases, exemplified by the inability to determine directionality for 75% of disease-associated genes due to limitations in GTEx expression quantitative trait loci models [60]. Future expansions in data diversity and analytical frameworks will enhance mechanistic understanding of LC-HF pathogenesis.

## Materials and methods

### Study populations

We collected GWAS datasets for 30 types of cancer from European populations, with each dataset containing more than 1 million SNVs. Summary statistics for the other 14 CVDs were derived from UK Biobank (https://www.ukbiobank.ac.uk/), FinnGen (https://www.finngen.fi/en) or numerous large consortia2,4–6. A summary of the GWAS cohorts used in this study, including study names, populations, and sample sizes, is provided in Table 1.

**Table 1:** Summary of GWAS cohorts used in this study

REAGENT or RESOURCE	SOURCE	IDENTIFIER
Pan-Cancer		
Acute myeloid leukemia	GCST90042758	10.1038/s41588-021-00954-4IF: 29.0 Q1 B1
Urinary bladder carcinoma	GCST90041857	10.1038/s41588-021-00954-4IF: 29.0 Q1 B1
Brain cancer	GCST90435625	10.1038/s41588-018-0184-yIF: 29.0 Q1 B1
Lobular carcinoma in situ	GCST90043925	10.1038/s41588-021-00954-4IF: 29.0 Q1 B1
Ductal carcinoma in situ breast cancer	GCST90079128	10.1038/s41586-021-04103-zIF: 48.5 Q1 B1
Invasive breast cancer	GCST90435602	10.1038/s41588-018-0184-yIF: 29.0 Q1 B1
Breast cancer	GCST90011804	10.1038/s41467-020-18246-6IF: 15.7 Q1 B1
Bile duct breast cancer	GCST90043859	10.1038/s41588-021-00954-4IF: 29.0 Q1 B1
Chronic lymphocytic leukemia	GCST90043911	10.1038/s41588-021-00954-4IF: 29.0 Q1 B1
Colon cancer	GCST90043851	10.1038/s41588-021-00954-4IF: 29.0 Q1 B1
Colorectal cancer	GCST90018808	10.1038/s41588-021-00931-xIF: 29.0 Q1 B1
Lung adenocarcinoma	GCST004744	10.1038/ng.3892IF: 29.0 Q1 B1
Small cell lung carcinoma	GCST004746	10.1038/ng.3892IF: 29.0 Q1 B1
Lung cancer(meta GWAS)	GCST004748	10.1038/ng.3892IF: 29.0 Q1 B1
Squamous cell Lung carcinoma	GCST004750	10.1038/ng.3892IF: 29.0 Q1 B1
Hodgkins Lymphoma	GCST90042738	10.1038/s41588-021-00954-4IF: 29.0 Q1 B1
Leukemia	GCST90042803	10.1038/s41588-021-00954-4IF: 29.0 Q1 B1
Liver cancer	GCST90041897	10.1038/s41588-021-00954-4IF: 29.0 Q1 B1
Lymphoma	GCST90042740	10.1038/s41588-021-00954-4IF: 29.0 Q1 B1
Major Salivary Gland Carcinoma	GCST90041792	10.1038/s41588-021-00954-4IF: 29.0 Q1 B1
multiple myeloma	GCST90042760	10.1038/s41588-021-00954-4IF: 29.0 Q1 B1
Non-Hodgkins lymphoma	GCST90042741	10.1038/s41588-021-00954-4IF: 29.0 Q1 B1
Oral cavity cancer	GCST90041793	10.1038/s41588-021-00954-4IF: 29.0 Q1 B1
Cancer of oropharynx	GCST90435573	10.1038/s41588-018-0184-yIF: 29.0 Q1 B1
Pancreatic cancer	GCST90041814	10.1038/s41588-021-00954-4IF: 29.0 Q1 B1
Prostate carcinoma	GCST90274714	10.1038/s41588-023-01534-4IF: 29.0 Q1 B1
Rectal cancer	GCST90043857	10.1038/s41588-021-00954-4IF: 29.0 Q1 B1
Cancer of major Salivary glands	GCST90041792	10.1038/s41588-021-00954-4IF: 29.0 Q1 B1
Cancer of tongue	GCST90435569	10.1038/s41588-018-0184-yIF: 29.0 Q1 B1
cardiovascular diseases		
stroke	METASTROKE collaboration	10.1038/ng.2480IF: 29.0 Q1 B1
Intracerebral hemorrhage	METASTROKE collaboration	10.1038/ng.2481IF: 29.0 Q1 B1
Hemorrhage stroke	GCST90297589	10.1186/s13073-023-01265-5IF: 11.2 Q1 B1
pulmonary embolism	GCST90077667	10.1038/s41586-021-04103-zIF: 48.5 Q1 B1
Aortic aneurysm	GCST90084034	10.1038/s41586-021-04103-zIF: 48.5 Q1 B1
Abdominal aortic aneurysm	GCST90084033	10.1038/s41586-021-04103-zIF: 48.5 Q1 B1
Thoracic aortic aneurysm	GCST90027266	10.1016/j.ajhg.2021.06.016IF: 8.1 Q1 B1
Coronary artery disease	CARDIoGRAMplusC4D	10.1038/ng.784IF: 29.0 Q1 B1
Atrial fibrillation	GCST006414	10.1038/s41588-018-0171-3IF: 29.0 Q1 B1
Heart failure	GCST009541	10.1038/s41467-019-13690-5IF: 15.7 Q1 B1
Heart failure	GCST90162626	10.1038/s41467-022-34216-6IF: 15.7 Q1 B1
Arterial hypertension	GCST007228	10.1016/S2213-2600(18)30409-0IF: 32.8 Q1 B1
Peripheral artery disease	GCST90018890	10.1161/CIRCGEN.119.002862IF: 5.5 Q1 B2
Myocardial infarction	GCST011364	10.1093/eurheartj/ehaa1040IF: 35.6 Q1 B1
Cardiomyopathy	GCST90296097	10.1038/s41467-023-43771-5IF: 15.7 Q1 B1

### Genome-wide genetic correlation

We computed genome-wide genetic correlation between traits using linkage disequilibrium (LD) score regression (LDSC). Briefly, it quantifies the separate contributions of polygenic effects by examining the relationship between LD scores and test statistics of SNVs from GWAS summary results, producing a genetic correlation based on the deviation of chi-square statistics from the null hypothesis. LDSC also applies a self-estimated intercept during the analysis to account for shared subjects between studies. The derived estimates range from − 1 to 1, with − 1 indicating a perfect negative genetic correlation and 1 indicating a perfect positive genetic correlation. We used pre-computed LD scores obtained from ~ 1 million common SNVs in the well-imputed HapMap3 European ancestry panel. A Bonferroni-corrected *P* value threshold of 0.0015 (0.05/32) was used to define statistical significance.

### Mendelian randomization (MR) analysis

The study employed five different Mendelian randomization (MR) methods: Weighted Median Regression, Inverse Variance Weighting (IVW), MR-Egger, Simple Mode, and Weighted Mode. Among these, the primary statistical model applied was IVW with random effects, which offers robust estimation of causal relationships between genetic variants and outcomes. To account for potential horizontal pleiotropy of instrumental variables (IVs), we utilized two advanced approaches: MR-Egger regression and the MR-Pleiotropy Residual Sum and Outlier (MR-PRESSO) method. The MR-PRESSO method is particularly valuable as it not only detects horizontal pleiotropy but also identifies and removes outliers among the IVs. Following the removal of these outliers, we repeated the MR-Egger and MR-PRESSO tests to ensure the consistency of the results.

To assess the heterogeneity across the IVs, we used Cochran’s Q statistic, which quantifies the variation in IV estimates and helps identify potential biases. Additionally, to verify the robustness and stability of our causal effect estimates, we conducted a leave-one-out sensitivity analysis. This analysis systematically excluded each SNV to identify any individual variant that might disproportionately influence the overall results. All MR analyses were performed using the R package TwoSampleMR(version 4.1.3).

### Cross-trait meta-analysis

To identify pleiotropic loci shared between two traits, we conducted a cross-trait meta-analysis of GWAS summary statistics using the Multi-Trait Analysis of GWAS (MTAG) method. MTAG employs generalized inverse-variance-weighted meta-analysis for multiple traits, and it effectively handles potential sample overlap between GWAS datasets. The underlying assumption of MTAG is that all single nucleotide variants (SNVs) share a consistent variance-covariance matrix of effect sizes across traits. As initially described, MTAG is a consistent estimator, and its effect estimates yield a lower genome-wide mean squared error compared to the corresponding single-trait GWAS estimates. Furthermore, MTAG provides stronger statistical power for detecting associations and minimizes false discovery rate (FDR) inflation, particularly for traits with high correlation. However, recognizing that the assumptions in MTAG—such as equal SNV heritability across traits and the same genetic covariance between traits—may not always hold true, we performed a sensitivity analysis using the cross-phenotype association analysis (CPASSOC) method. CPASSOC integrates GWAS summary statistics from multiple traits to detect shared variants while adjusting for population structure and cryptic relatedness. It provides two test statistics: SHom and SHet. SHom is derived from a fixed-effect meta-analysis and can be interpreted as the maximum weighted sum of trait-specific genetic effects. While useful, SHom may lack power when there is between-study heterogeneity, which is common in meta-analyses involving multiple traits. To address this, we adopted SHet, an extension of SHom that improves power by accommodating heterogeneous effects resulting from differences in study designs, environmental factors, populations, or phenotypes—common in practical applications. To ensure the selection of independent SNVs, we performed PLINK clumping with the following parameters: --clump-p1 5 × 10^− 8^, --clump-p2 1 × 10^− 5^, --clump-r2 0.1, and --clump-kb 1000. Significant pleiotropic SNVs were defined as variants that met the criteria of having P values below 5 × 10^− 8^ in both the individual GWAS studies and in the meta-analysis (i.e., PMTAG and PCPASSOC). For functional annotation of the variants identified through MTAG and CPASSOC, we used the ANNOVAR tool.

### Colocalization analysis

We used the R package coloc to determine whether the association signals for lung carcinoma and heart failure traits co-localized. For 48 shared SNVs between traits, we extracted the variants within 500 kb of the index SNV and calculated the probability that the two traits share one common causal variant (H4). Loci with a probability greater than 0.5 were considered to colocalize.

### SNV phenotype analysis

To investigate trait pleiotropy and evidence of association of urinary trait loci with other traits, we extracted from the GWAS Catalog pleiotropic loci shared between two traits at *P* < 5 × 10^− 8^.

### Metabolomics analysis

Metabolomics analysis was performed using plasma samples from the METSIM study52, which included 6,136 Finnish male participants and 1,391 plasma metabolites (*p*-value < 10^ (−6)). Additionally, using the mGWAS-Explorer53 API (https://www.mgwas.ca), which integrates data from 65 European population-based mGWAS databases, no significant associations (*p* < 1 × 10^(−5)) were found between various metabolites and genetic variants.

### GTEx tissue specific expression analysis

We evaluated all genome-wide significant SNVs for evidence of eQTL using the GTEx database(www.gtexportal.org). We additionally searched median gene expression levels in 53 tissues from the GTEX database and standardized gene expression values to map tissue-specific and cell-specific expression of genes near and/or in eQTL (eGenes). All analyses are based on R package xQTLbiolinks.

### Gene-based association analysis

We employed three distinct methods—TWAS-Fusion, SMR, and MAGMA—to identify genes shared between lung carcinoma and HF traits. The input files for all gene-level analyses were the complete GWAS summary statistics derived from the MTAG meta-analysis. In each method, the p-value threshold was adjusted using Bonferroni correction to control for multiple comparisons.

TWAS (Transcriptome-Wide Association Study) identifies tissue-specific gene-trait associations by integrating GWAS data with cis-SNV-based gene expression models. We performed TWAS using the FUSION software, leveraging post-mortem tissue expression profiles from GTEx (version 6), which includes pre-computed models.

Summary-data-based Mendelian Randomization (SMR) analysis combines GWAS data with eQTL studies to pinpoint genes whose expression levels are associated with complex traits due to either pleiotropy or causality. A significant SMR association could indicate a causal relationship, where the causal variant influences disease risk through changes in gene expression, pleiotropy, where the causal variant has pleiotropic effects on both gene expression and disease risk, or linkage, where separate causal variants affect gene expression and disease. To distinguish pleiotropy from linkage, SMR employs the HEIDI-outlier test. In our analysis, we utilized cis-eQTL summary data for whole blood from eQTLGen, a meta-analysis of 31,684 blood samples, as well as from GTEx V8 for nine relevant tissues, including artery aorta, subcutaneous adipose, artery coronary, artery tibial, heart atrial appendage, heart left ventricle, kidney cortex, liver, and whole blood. Genes significantly associated with lung carcinoma and HF trait pairs were defined by passing the Bonferroni-corrected *p*-value thresholds and having a PHEIDI value greater than 0.05.

MAGMA (Multi-marker Analysis of GenoMic Annotation) is a robust method that employs multiple regression to properly account for linkage disequilibrium (LD) between markers and detect multi-marker effects. We conducted MAGMA analysis using the default settings and the European ancestry panel from the 1000 Genomes Project (Phase 3) as the LD reference.

### Over-representation enrichment analysis

We used the “clusterProfiler” package to perform GO (Gene Ontology) and KEGG (Kyoto Encyclopedia of Genes and Genomes) pathway enrichment analysis on the genes. Benjamini–Hochberg procedure was used to account for multiple tests (FDR < 0.05).

### Single cell analysis

The publicly available and in-house FASTQ files generated by 10× Genomics were aligned and quantified against the GRCh38 human reference genome using Cell Ranger software (Version 6.1.2) with default settings. The output from Cell Ranger and the count matrix were processed using the Read10X function from the Seurat package (Version 4.0.4) and the read.table function, respectively. The count matrix was then converted to the dgCMatrix format for further analysis. To eliminate potential interference from doublets, we removed predicted doublets using Scrublet. The merge function was employed to integrate individual datasets into a unified object, and the RenameCells function was used to ensure that all cell labels were unique. In total, 269,794 cells from various studies were pooled for analysis. Quality control was conducted based on several criteria. Cells with fewer than 200 detected genes or more than 20% mitochondrial content were excluded. Additionally, cells with over 6,000 detected genes were removed to further minimize the presence of doublets. To normalize the data, we used the global-scaling method “LogNormalize,” ensuring that the total gene expression for each cell was standardized with a scale factor of 10,000. The top 2,000 most variable genes were selected for downstream analysis using the FindVariableFeatures function. To regress out unwanted sources of variation, we used the ScaleData function, specifying UMI count and mitochondrial percentage as variables to regress. Principal component analysis (PCA) was performed on the highly variable features to reduce dimensionality, with the first 30 principal components selected for further analysis. To address batch effects, we applied the RunFastMNN function from the SeuratWrappers package (Version 0.3.0) for batch correction. Clustering was performed based on the edge weights between pairs of cells, using a shared nearest-neighbor graph generated by the Louvain algorithm implemented in the FindNeighbors and FindClusters functions. The resulting clusters were visualized using the UMAP method. For subclustering, a similar approach was applied, including normalization, selection of variably expressed features, dimensionality reduction, batch correction using RunFastMNN, and clustering. To annotate the cell clusters, differentially expressed markers were identified using the FindAllMarkers function, applying the default nonparametric Wilcoxon rank-sum test with Bonferroni correction.

### Drug target analysis

We utilized the following four drug databases: DrugCentral is a comprehensive drug information resource that integrates data from multiple sources, offering detailed information on drug properties. DGIdb is a database that focuses on drug-gene interactions, providing information on how drugs interact with specific genes, which can be used to identify potential drug targets and explore drug repurposing opportunities based on genetic interactions. PharmGKB is a pharmacogenomics database that compiles data on genetic variations and their impact on drug response. DrugBank is a comprehensive resource that combines detailed information on FDA-approved drugs, investigational drugs, and their targets, offering insights into drug mechanisms, therapeutic potential, interactions, and pharmacokinetic properties.

## Conclusion

We performed integrative analyses of large-scale datasets to investigate the genetic interplay between pan-cancers and CVDs. We identified the most significant genetic association between LC and HF, although a causal relationship between the two conditions was not established. Furthermore, our analyses uncovered key target genes and pathways implicated in their shared etiology. These target genes were further validated at the transcriptomic level using single-cell sequencing, and concurrently, we identified potential therapeutic agents for managing these comorbidities through drug repurposing analyses. Collectively, our findings elucidate the genetic basis underlying the clinically observed co-morbidity of LC and HF, and propose targeted therapeutic strategies that may ultimately improve patient prognosis.

## Supplementary Information

Supplementary Material 1.

Supplementary Material 2.

Supplementary Material 3.

Supplementary Material 4.

Supplementary Material 5.

## Data Availability

This paper analyzes publicly available datasets, which are detailed in the Supplementary Data table. Cancer-related data were obtained from the publicly accessible GWAS Catalog dataset, as listed in Supplementary Data 1. Cardiovascular data were retrieved from publicly available cardiovascular disease GWAS databases, also presented in Supplementary Data 1.All codes supporting the conclusions of this article will be made available by the authors, without undue reservation.

## Abbreviations

LC: Lung Cancer
HF: Heart Failure
GWAS: Genome-Wide Association Study
SNV: Single Nucleotide Variant
MTAG: Multi-Trait Analysis of GWAS
CPASSOC: Cross-Phenotype Association Analysis
LDSC: Linkage Disequilibrium Score Regression
eQTL: Expression Quantitative Trait Locus
